# Insights Into the Pathophysiology of Catecholamine-Refractory Shock: A Narrative Review

**DOI:** 10.7759/cureus.86224

**Published:** 2025-06-17

**Authors:** Ana Gonçalves, Filipa Gonçalves Pereira, Susana Fernandes, João Gonçalves Pereira

**Affiliations:** 1 Intensive Care Unit Department, Hospital Vila Franca de Xira, Vila Franca de Xira, PRT; 2 Critical Care University Clinic, Faculdade de Medicina Universidade de Lisboa, Lisboa, PRT; 3 Intensive Care Department, Hospital de Santa Maria, Lisbon, PRT

**Keywords:** catecholamine-refractory shock, critical care, norepinephrine, septic shock, vasoplegia, vasopressin, vasopressor resistance

## Abstract

Shock, characterized by severe hemodynamic failure and tissue hypoperfusion, is a life-threatening condition that requires immediate recognition and adequate treatment. Some patients exhibit a poor response to catecholamines, progressing to refractory shock, and have a high mortality risk. We aimed to review the characteristics of patients associated with the development of refractory shock and to evaluate proposed strategies for improving prognosis. Refractory shock remains poorly defined due to unclear pathophysiology. The failure of mitochondria to produce energy, neurohormonal dysregulation, adrenergic receptor desensitisation, and inflammatory vasodilation all contribute to this condition. Prompt recognition of at-risk patients is essential and may be supported by clinical signs, vasopressor load, and biomarkers such as lactate and base excess. Multimodal strategies, which combine vasopressors with complementary mechanisms, corticosteroids, and metabolic support, present a promising approach to enhance outcomes. Further research is required to refine shock phenotyping and guide personalised therapy.

## Introduction and background

Shock is a life-threatening condition caused by acute circulatory failure, in which inadequate tissue perfusion leads to widespread cellular hypoxia, organ dysfunction, and, if unresolved, death [1]. It fundamentally results from the inability of cardiac output to meet the metabolic demands of vital organs.

Maintaining adequate cardiac output is crucial for ensuring sufficient perfusion and supporting the function of cells and organs. Consequently, cardiac output must adapt dynamically to meet tissue demands, which is achieved through changes in venous return. The inability of the cardiovascular system to maintain sufficient cardiac output and systemic perfusion underlies all shock states, regardless of aetiology [2].

Shock is traditionally classified into four main hemodynamic profiles: hypovolemic, cardiogenic, obstructive, and distributive. Regardless of the profile, and while the underlying cause of shock is resolved, perfusion pressure is secured through optimization of venous return and noradrenaline administration. Most frequently, distributive shock, particularly septic shock, associated with profound vasodilation and reduced vascular tone, evolves into catecholamine-refractory shock [2]. Catecholamine-refractory shock results from the persistence of hypotension and signs of hypoperfusion despite adequate fluid resuscitation and high-dose catecholamine support, most commonly norepinephrine ≥ 0.25-1 μg/kg/min [3]. This condition reflects not only vascular hyporesponsiveness but also complex pathophysiological processes, such as adrenergic receptor downregulation, nitric oxide (NO)-mediated vasodilation, mitochondrial dysfunction, and disruption of the hypothalamic-pituitary-adrenal axis [4].

Escalating doses of noradrenaline are independently associated with increased mortality, arrhythmias, myocardial stress, and immunosuppressive effects [5]. Hence, a multimodal approach has been advocated, integrating non-adrenergic vasopressors (e.g., vasopressin, angiotensin II, methylene blue), corticosteroids, metabolic adjuncts, and advanced hemodynamic monitoring, to tailor therapy and minimise vasopressor load [6].

Emerging biomarkers such as lactate and base excess (BE) have been explored, not only as indicators of disease severity but also as potential tools for the early identification of patients at risk of developing catecholamine-refractory shock. Lactate elevation is part of a complex metabolic adaptation to stress, while BE may more accurately reflect mitochondrial energy failure and systemic acidosis [7].

This narrative review aims to synthesize current evidence on catecholamine-refractory shock, examining its definitions, pathophysiology, clinical markers, and evolving therapeutic strategies, with particular focus on individualized vasopressor use and timely recognition of refractory states.

## Review

Pathophysiology of shock

Several factors can lead to cardiac output failure, causing inadequate perfusion to meet organ demands [8]. These factors result in distinct hemodynamic changes, which have been categorized into four major hemodynamic profiles: hypovolemic, cardiogenic, distributive, and obstructive shock. Each one exhibits a distinct mechanism that leads to cardiac output failure.

Classification of shock

Hypovolemic shock results from inadequate intravascular volume, leading to reduced preload. Cardiogenic shock occurs when the primary cardiac pump fails, resulting in the heart's inability to generate sufficient output despite adequate filling pressures. When the heart fails to function effectively as a pump, cardiac output and blood pressure decrease, resulting in inadequate tissue perfusion that ultimately can lead to shock [9]. It primarily refers to the loss of the ability to relax or the accumulation of telesystolic volume (due to systolic failure), which precludes receiving all the venous return. Distributive shock is caused by severe vasodilation and loss of vascular tone, associated with a reduction in stress volume, leading to decreased venous return. This, in turn, lowers cardiac output and impairs tissue perfusion, ultimately resulting in shock [10]. Patients often have wide pulse pressure and initially high or normal cardiac output, but experience inadequate perfusion at the cellular level. Obstructive shock refers to an acute obstruction of blood flow at a central level, impairing cardiac filling or output despite normal cardiac function [11]. All these pathophysiological conditions result from the cardiac output not meeting cellular requirements, leading to shock.

Catecholamine-Refractory Shock

The “final common pathway” of prolonged shock is cellular hypoxia and inflammation, which perpetuates vasodilation, depresses cardiac function, changes microcirculation and cellular perfusion, irrespective of the initial cause [12]. Catecholamine-refractory shock thus represents a point when standard therapy (fluids and catecholamines) fails because the underlying pathophysiology has become multifactorial and self-sustaining.

**Figure 1:**
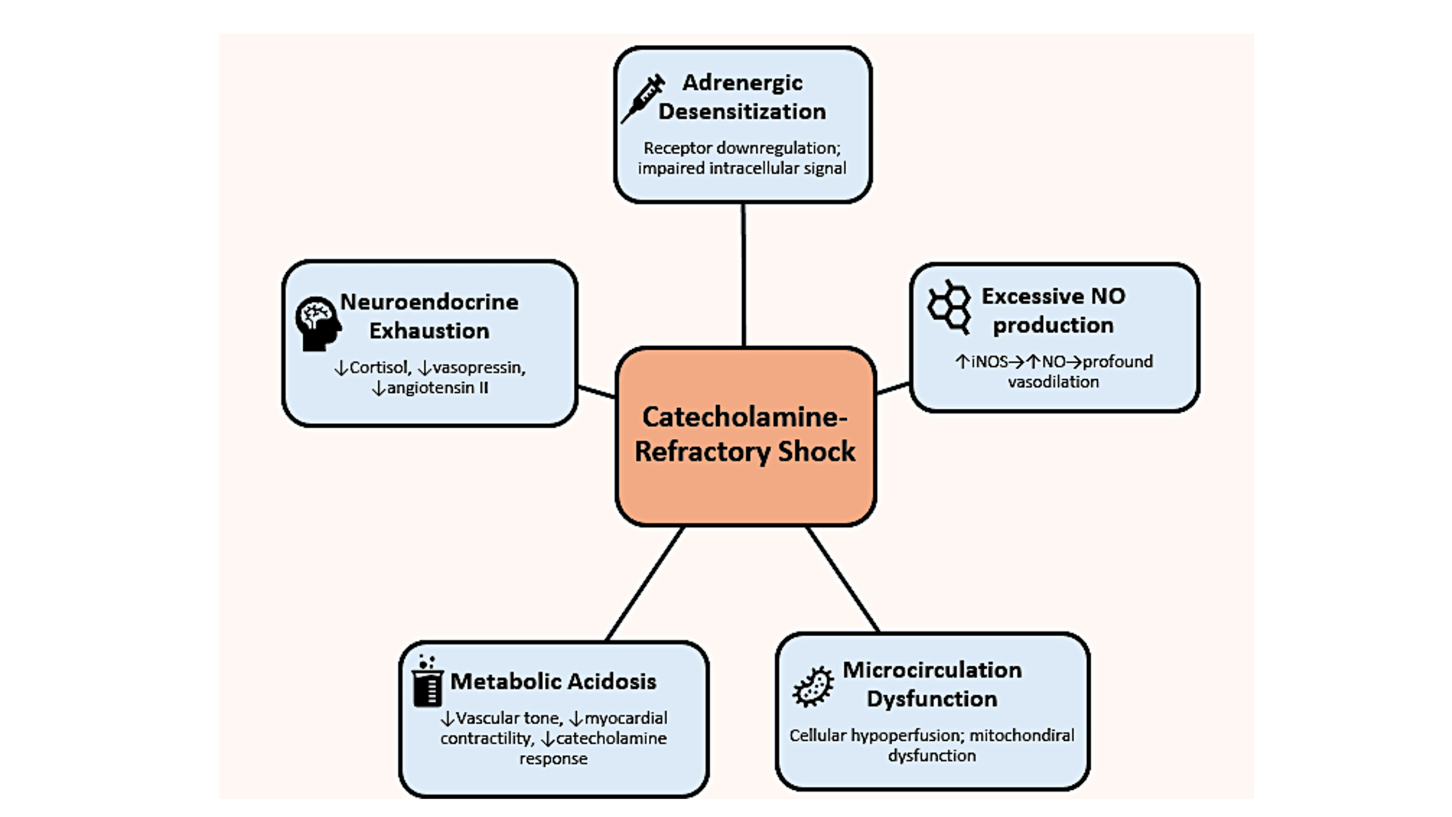
Pathophysiology of catecholamine-refractory shock NO - Nitric oxide; iNOS - Inducible nitric oxide synthase Image credit: Authors

Catecholamine-refractory shock is characterized by vascular hyporesponsiveness - the failure of blood vessels to constrict appropriately, despite high levels of endogenous or exogenous catecholamines [13]. Multiple mechanisms underlie this refractory vasoplegia (Figure 1).

Adrenergic receptor desensitization: Prolonged exposure to high catecholamine levels leads to downregulation and desensitization of adrenergic receptors on vascular smooth muscle. In septic shock, α-1 adrenergic receptors can be internalized or uncoupled from signalling pathways, reducing the vasoconstrictive effect of norepinephrine. β-adrenergic receptors may also become desensitized, contributing to myocardial depression and reducing the inotropic response [14]. High-dose catecholamines can disrupt intracellular signalling (e.g., calcium handling in vascular cells), further blunting contractility. Using multiple vasopressors with different mechanisms can help mitigate single-receptor tachyphylaxis [4].

Excess NO and vasodilatory mediators: An uncontrolled inflammatory response induces inducible NO synthase (iNOS), causing excessive NO production. NO stimulates guanylate cyclase, increasing cyclic guanosine monophosphate (cGMP) in vascular smooth muscle and causing profound vasodilation and loss of vascular tone. In refractory shock, this pathologic vasodilation persists and impairs catecholamine signalling. Other vasodilatory mediators, such as prostaglandins and bradykinin, may also contribute. Agents that counteract the NO-cGMP pathway (e.g., methylene blue) can transiently reverse vasoplegia by inhibiting iNOS and guanylate cyclase [14].

Microcirculatory dysfunction: Refractory shock involves dysregulation at the microcirculation and cellular level. In sepsis, blood flow distribution becomes heterogeneous - some capillary beds receive insufficient flow even when global hemodynamics seem restored [15]. This maldistribution of blood flow leads to regions of cellular hypoperfusion despite normal or high cardiac output, a phenomenon sometimes called “cryptic shock” [16]. The underlying mechanisms include endothelial injury, glycocalyx degradation, leukocyte adhesion, and formation of microthrombi [4]. Mitochondrial dysfunction emerges due to oxidative and inflammatory stress, impairing ATP synthesis. Multiple mechanisms are implicated, including inhibition of the pyruvate dehydrogenase complex, disruption of oxidative phosphorylation, and excessive generation of reactive oxygen species [6].

Metabolic derangements and acidosis: Severe acidosis commonly complicates shock. Acidosis impairs catecholamine-receptor binding and signal transduction, although the same occurs at extreme pH deviations [17]. Additionally, adenosine triphosphate (ATP) depletion compromises vascular tone and myocardial contractility. The energy expenditure, which complicates prolonged hypoperfusion, further contributes to acidosis through the release of H+ from the ATP [18]. Hypocalcaemia also occurs in critical illness and can depress myocardial and vascular contraction; calcium is essential for excitation-contraction coupling. If ionized calcium is very low, vasopressors may be less effective [19]. Paradoxically, parenteral calcium administration may be associated with increased mortality [20].

Neuroendocrine exhaustion: Prolonged shock states can exhaust or overwhelm the body’s stress hormone responses [21]. Absolute or relative deficiencies of endogenous vasoactive hormones, such as cortisol, vasopressin (antidiuretic hormone), and angiotensin II, frequently occur in vasodilatory shock [13]. In septic shock, critical illness-related corticosteroid insufficiency (CIRCI) is frequent, affecting up to 50% of patients. CIRCI reflects inadequate cortisol synthesis or peripheral resistance, impairing vascular tone and inflammation control [22]. Cortisol is required to maintain adrenergic receptor expression and signal transduction. Similarly, vasopressin (antidiuretic hormone) concentration rises in the early state of septic shock but may fall paradoxically after prolonged sepsis. Low plasma angiotensin II concentration has also been noted in refractory shock, possibly due to receptor upregulation or excessive consumption. These hormonal deficits explain why supplementation (hydrocortisone, vasopressin, or angiotensin II) can restore responsiveness.

Clinical identification and diagnostic challenges

Early identification of catecholamine-refractory shock is critical, as delays in appropriate intervention are associated with a dismal prognosis. Progressing to refractory shock should be suspected when increasingly high-dose vasopressors are required to maintain blood pressure, along with persistent signs of hypoperfusion. Key considerations in identification and diagnosis include the following:

Hemodynamic criteria: There is no single universal threshold defining refractory shock, but most definitions involve an inability to reach a target mean arterial pressure, despite high vasopressor doses. The need for a second or third vasopressor agent, such as adding vasopressin to norepinephrine, is a practical indicator.

An important issue is the difference in relative potency, related to the norepinephrine base, of the various norepinephrine salts available [23], which influences the definition itself. This, together with different ICU standards of care, might lead to the addition of a second or third vasopressor at different noradrenaline doses or in various clinical situations.

Vasopressor load: The vasopressor load refers to the total catecholamine dose burden. Studies have found that higher vasopressor requirements are strongly associated with myocardial toxicity and mortality in shock [24]. Importantly, high-dose vasopressors (e.g., norepinephrine >1 μg/kg/min) often correlate with impending multisystem organ failure [25].

Clinical signs of hypoperfusion: Physical exam and bedside monitoring provide early clues. Signs of inadequate perfusion include altered mental status, cool mottled extremities, oliguria, and prolonged capillary refill. In refractory shock, these signs persist or worsen despite initial fluid resuscitation and vasopressor therapy.

Biomarkers

Lactate

Lactate is widely used as an indicator of hypoperfusion and as a marker for hemodynamic resuscitation. In shock, lactate levels typically rise. Common misconceptions attribute its rise to a supposed “deficit of oxygen” and anaerobiosis [6]. However, eukaryotic cells do not use anaerobic metabolism to produce energy. In the absence of oxygen (ischemia), but not during hypoxia, mitochondrial apoptosis is noted [26], and cells die. Paradoxically, during multiorgan failure, clinical and biochemical failure is present, yet there is minimal cell death, tissue oxygenation is maintained, and there is an almost full recovery in survivors [27].

Lactate is produced from pyruvate and two hydrogen ions (a reduction reaction) and, consequently, increases intracellular pH. Pyruvate accumulation and metabolism result from the blocking of the pyruvate complex dehydrogenase in the mitochondrial membrane, a response to adrenaline release, in stressful situations. The reduction of pyruvate into lactate protects the cell from the acidic environment [28]. Lactate is then released into the circulation and absorbed by the brain and the heart, providing a rapid source of energy to these organs, essential for this stress reaction, the “fight or flight response” [29]. Thus, elevated lactate is essentially a marker of illness severity and stress response, and not of hypoxia or anaerobiosis.

Base excess and metabolic acidosis

Base excess is calculated from arterial blood gas and reflects the metabolic component of the acid-base status. A significantly negative BE (e.g., ≤ -6 mmol/L) indicates metabolic acidosis, due to unmeasured anion accumulation in shock, including ketones, toxins, and strong ion shifts. BE correlates with the degree of acid load and serves as a surrogate marker of global tissue hypoperfusion and ATP hydrolysis. The combination of BE and lactate provides complementary insights [30]: for instance, a high lactate with near-normal BE may reflect adrenergic stress, whereas a high lactate with a large base deficit strongly suggests impending cellular energy failure and severe shock [31]. Early in refractory shock, a significantly negative BE (indicating high metabolic acidosis) is associated with worse prognosis [32].

Early identification of catecholamine-refractory shock allows timely deployment of adjunctive therapies (additional vasopressors, corticosteroids, etc.) and consideration of advanced monitoring and support before irreversible damage occurs.

Several candidate biomarkers are under investigation for stratifying shock severity, especially plasma renin and vasopressin concentration. Immune phenotyping may also play a role as a complementary approach, such as elevated IL-10, reduced HLA-DR expression, or lymphopenia [33]. Their integration into clinical practice could support personalized resuscitation strategies and more targeted use of emerging vasopressors.

Prognostic markers

Several prognostic markers have been assessed to provide early identification of the most severe patients, with a high mortality risk. This approach may help in the early identification of patients who might benefit from early ICU admission.

In a recent single-centre study, six different biomarkers were evaluated, including azurocidin (AZU1), soluble triggering receptor expressed on myeloid cells (sTREM), soluble urokinase-type plasminogen activator receptor (suPAR), high-sensitivity C-reactive protein (hsCRP), procalcitonin (PCT), and interleukin-6 (IL-6), for predicting mortality. suPAR had the highest area under the curve, 0.81 (95% CI: 0.67-0.91), with a cutoff value > 8,168 ng/mL [34].

Management strategies

Because shock is a life-threatening and time-sensitive condition, early recognition and prompt treatment are critical for survival. During the initial phase of shock, rapid fluid infusion is used to improve venous return and increase cardiac output - the main driver of tissue perfusion [35]. However, only 50-60% of patients respond to fluids early on, and up to 25% may become non-responders after initial resuscitation [36]. Therefore, assessing fluid responsiveness before giving large fluid volumes is essential to avoid fluid overload. The same is particularly important when cardiogenic or obstructive shock is suspected, as, in these situations, increasing venous return does not improve cardiac output.

If fluids fail to improve cardiac output or if tissue perfusion remains poor despite an adequate cardiac output, a prompt reassessment of the circulatory status is needed. Additionally, echocardiography is helpful to evaluate cardiac function and guide treatment [37]. Direct monitoring of cardiac output and hemodynamic parameters may also help to select appropriate therapy [38].

Vasopressors

Alongside fluid replacement therapy, vasopressors should be administered to restore hemodynamic status (Table 1), especially if vasoplegia is suspected. They act by increasing vascular tone in both venous and arterial territories: veno-constriction to promote the increase of the venous return, and arterial-constriction to increase blood and perfusion pressures. Preventing or decreasing the duration of hypotension has been associated with better outcomes in patients with vasodilation [39]. Norepinephrine is the first-choice vasopressor in septic and vasodilatory shock and should be started early in patients who are not responding to fluids and remain hypotensive or with signs of hypoperfusion [40] (Table 1).

**Table 1:** Vasopressors and adjuvants in catecholamine-refractory shock AT1 - Angiotensin 1

Drug	Mechanism	Indications	Adverse Effects	
Norepinephrine	α1 (vasoconstriction), mild β1	First-line for septic/vasodilatory shock	Arrhythmias, myocardial toxicity	[41,42]
Vasopressin	V1 (vasoconstriction), V2 (renal water reabsorption)	Added to norepinephrine in refractory septic shock	Digital and mesenteric ischemia	[43,44]
Terlipressin	V1 analogue with longer half-life	Refractory shock (limited use due to side effects)	High risk of ischemia	[45]
Epinephrine	α1 and β1 adrenergic agonist	Anaphylaxis; rescue agent in septic/cardiogenic shock	Tachyarrhythmias, increased lactate	[46]
Angiotensin II	AT1 receptor agonist (vasoconstriction)	Refractory vasodilatory shock	Thromboembolism, excessive vasoconstriction	[47]
Methylene Blue	Inhibits Nitric oxide synthase and soluble guanylate cyclase	Severe vasoplegia, post-cardiopulmonary bypass	Pulmonary hypertension, cyanosis	[48]
Dobutamine	β1-agonist (inotropic), some β2 effect	Low cardiac output shock (cardiogenic/septic)	Tachycardia, arrhythmias	[49]
Milrinone	Phosphodiesterase 3 inhibitor, increases cyclic adenosine monophosphate	Inotropy in β-blocked or unresponsive patients	Hypotension, may require vasopressors	[50]
Levosimendan	Calcium sensitizer, K+ channel opener	Rescue therapy in refractory cardiogenic shock	Long duration, hypotension	[51]
Corticosteroids	Glucocorticoid receptor agonist	Adjunct in septic shock (controversial)	Hyperglycemia, immunosuppression	[52,53]
Ascorbic Acid	Antioxidant and endothelial-protective	Investigational use in shock (not routinely recommended)	No proven benefit, risk of oxalate nephropathy	[54]
High-dose Insulin	Overcoming of insulin-resistance; Improvement of coronary blood flow	Low cardiac output shock	Hypokaliemia; Hypoglicemia	[55]

Norepinephrine: It is a catecholamine released by the sympathetic nervous system that acts primarily on α1 adrenergic receptors, causing potent vasoconstriction. Other effects include moderate β1 adrenergic activity, leading to a modest increase in cardiac contractility, stimulation of renin secretion from the kidneys, pupil and bronchiole dilation, and gastrointestinal peristalsis inhibition, responses consistent with the classic “fight or flight” state [41]. Despite norepinephrine being effective in restoring perfusion pressure, high doses are associated with significant toxicity and poor outcomes (Table 2).

**Table 2:** Organ-specific effects of norepinephrine in shock states

Target Organ/System	Physiological Effect of Norepinephrine	Mechanism	Reference
Vascular	Potent vasoconstriction (arterial and venous)	α₁ adrenergic receptor activation → ↑ systemic vascular resistance	[40,41]
Kidneys	Stimulation of renin release Increase in renal blood flow	β₁ adrenergic receptor activation in juxtaglomerular cells. Increase in systemic blood pressure, and decreased renal sympathetic tone.	[41,56]
Lungs	Mild bronchodilation	β₂ adrenergic effects (less prominent)	[41]
Eyes	Pupil dilation (mydriasis)	α₁-mediated contraction of dilator pupillae muscle	[57]
Gastrointestinal Tract	Decreased motility and peristalsis	Sympathetic inhibition of acetylcholine release	[58]
Metabolism and Liver	↑ Glycogenolysis, ↓ insulin secretion	β adrenergic mediated metabolic response	[41]
Immune System	Immunosuppression at high doses	Catecholamine-induced modulation of cytokine production	[5,59]
Myocardium	Arrhythmias, myocardial stress	Dose-dependent β₁ overstimulation and increased oxygen demand	[5,25]
Microcirculation	Maldistribution of perfusion	Excessive vasoconstriction and endothelial dysfunction	[15]

The criteria for initiating a second vasopressor vary between centers, although recommendations for an early start are increasingly common [60].

Vasopressin: It is a non-catecholaminergic vasopressor that acts primarily on V1 receptors in vascular smooth muscle to induce vasoconstriction and on V2 receptors in the kidneys to promote water reabsorption. The VASST [61] and VANISH [62] trials found no significant mortality reduction with vasopressin compared to norepinephrine alone in unselected patients, but a post-hoc analysis indicated potential benefit in less severe cases, along with a norepinephrine-sparing effect [61]. The combination of norepinephrine and vasopressin appears particularly beneficial in septic shock [63], and an early start of combination therapy has been recommended to improve efficacy and help reduce mortality [45,64]. Unlike catecholamines, vasopressin does not induce tachyarrhythmias and may even reduce heart rate via reflex bradycardia, improving diastolic filling, although at higher doses, adverse events have been documented (digital, mesenteric, or cardiac ischemia) [14].

Terlipressin: As a synthetic analogue of vasopressin, it has a longer half-life and greater vascular selectivity. It is not presently recommended due to its higher risk of adverse events, mainly ischemic, and a potentially lower efficacy than vasopressin [65].

Epinephrine is a potent mixed α1 and β1 adrenergic agonist, offering both strong vasoconstriction (via α1 receptors) and cardiac inotropic stimulation (via β1 receptors). It is the first-line treatment for anaphylactic shock, where β effects promote bronchodilation and reduce mediator release, while α1 vasoconstriction counteracts vasodilation and edema [46]. In cardiogenic shock, epinephrine may be used as a rescue therapy, although it may induce tachyarrhythmias, especially at high doses [49]. In septic shock, epinephrine is often used as a second-line agent, particularly when norepinephrine and vasopressin are insufficient or unavailable. Given its pharmacological profile, epinephrine is generally reserved for scenarios requiring both inotropic and vasopressor support.

Angiotensin II: It is the final effector peptide of the renin-angiotensin-aldosterone system, exerting potent vasoconstrictive effects via AT1 receptors, a non-adrenergic mechanism of rapid onset, increasing systemic vascular resistance, and raising blood pressure [47]. It also promotes aldosterone secretion, enhancing sodium and water retention. It is mainly approved for refractory vasodilatory shock. It can also cause excessive vasoconstriction and thrombotic events.

Although it is still not clear which patients might benefit the most from angiotensin II, evidence suggests that patients with distributive shock, with persistent hypotension (mean arterial pressure below 65 mmHg), despite norepinephrine and fluid resuscitation, with no cardiac dysfunction, are the preferred candidates [66].

Methylene blue: It is a soluble guanylate cyclase inhibitor that counteracts NO-mediated vasodilation. A recent review by Arias-Ortiz et al. [48] summarizes methylene blue’s effects: typically, a transient increase in arterial pressure and systemic vascular resistance, but without a clear improvement in survival. Its effects are more evident when high NO concentration is expected, especially in septic shock and after cardiopulmonary bypass. Elevated pulmonary vascular resistance is a common adverse event.

Inotropic and adjuvant therapy

Dobutamine: As a β1 adrenergic agonist with some β2 activity, it is the first-line inotrope for cardiogenic shock and septic shock with low cardiac output, despite adequate fluid resuscitation. It increases stroke volume and heart rate. By improving cardiac output, dobutamine can enhance tissue perfusion. In septic shock patients who remain hypoperfused despite adequate blood pressure and blood volume, adding dobutamine is recommended [44].

Milrinone: It is a phosphodiesterase-3 inhibitor that increases intracellular cyclic adenosine monophosphate (cAMP), independently of β-receptors. It promotes inotropy and vasodilation, reducing both systemic and pulmonary vascular resistance [50]. It is often chosen in patients receiving β-blockers or with downregulated β-receptors, where dobutamine may be less effective. However, due to its vasodilatory profile, milrinone may worsen hypotension and often requires concomitant use of a vasopressor. In the DOREMI trial, there were no significant differences in outcomes between milrinone and dobutamine in patients with cardiogenic shock [50].

Levosimendan: As a calcium sensitizer and ATP-sensitive potassium channel opener, it improves contractility without increasing myocardial oxygen consumption [51]. It also induces vasodilation, reducing preload and afterload. Levosimendan is considered a third-line agent, often used in β-blocked patients or as a rescue therapy in refractory cardiogenic shock. Its long half-life (circa 80 hours via an active metabolite) means its effects persist well beyond infusion, which can be useful or problematic in unstable patients.

Corticosteroids: They have shown potential to improve mortality rates and physiological outcomes in patients with septic shock. Studies have demonstrated the role of corticosteroids in reducing organ failure, inflammatory markers, the need for organ-supporting interventions, and peripheral perfusion [52]. However, its use remains controversial [67], possibly due to its well-recognized adverse effects. Potential benefits may be attributed to the modulation of the sepsis-associated dysregulation of the hypothalamic-pituitary-adrenal axis, alterations in cortisol metabolism, and tissue resistance to glucocorticoids, known as relative adrenal CIRCI [68]. Changes in the inflammation profile or the increase in the vascular tone in response to catecholamines may also contribute to a benefit [52].

Ascorbic acid (vitamin C): Either alone or in conjunction with thiamine and corticosteroids, it has been suggested to play a role in the management of shock, due to its antioxidant, anti-inflammatory, and endothelial-protective properties. However, studies have not consistently demonstrated a benefit, and its use cannot be presently recommended [54].

High-dose insulin: Associated with glycose and potassium, it has been proposed as a “metabolic” strategy to address shock with a low cardiac output. Several mechanisms have been postulated, namely, the overcoming of relative insulin-resistance, the promotion of oxidative phosphorylation and vasodilation via the L-arginine-NO-pathway with improvement of coronary blood flow [55].

Limitations

In this narrative review, we aim to address the significant topic of catecholamine-refractory shock. We searched the relevant literature for recent reviews or meta-analyses focusing on its pathophysiology, therapeutics, primarily vasopressors, and diagnosis and prognosis. We also examined lactate and BE, discussing their metabolism, potential use as biomarkers, and the impact of various therapies on their concentrations. Furthermore, we systematically reviewed the bibliography of the referenced articles and the authors' databases for pertinent studies.

We acknowledge that this was a very comprehensive topic and that several important papers may have been overlooked. Furthermore, due to its significance, numerous new papers are consistently being published, and we may have missed some significant developments.

## Conclusions

Catecholamine-refractory shock remains associated with a high mortality rate. Controversy exists regarding its definition. Several mechanisms probably contribute to this resistance, although it is unknown if their identification and direct treatment improve prognosis. Additionally, increasing the dose of catecholamines may worsen prognosis by promoting arrhythmia, myocardial toxicity, and receptor desensitization. Evidence suggests that early addition of a second vasopressor (mainly vasopressin) to catecholamines may improve prognosis.

Early recognition and prompt, effective treatment rely mostly on clinical features. The use of microcirculation monitoring and biomarkers has the potential to facilitate early adoption of multimodal vasopressor strategies to counteract vasoplegia, combining vasopressors with different targets to support perfusion while limiting toxicity.
